# Comparison of audio vs. audio + video for the rating of shared decision making in oncology using the observer OPTION^5^ instrument: an exploratory analysis

**DOI:** 10.1186/s12913-018-3329-x

**Published:** 2018-07-04

**Authors:** Michael R. Gionfriddo, Megan E. Branda, Cara Fernandez, Aaron Leppin, Kathleen J. Yost, Brittany Kimball, Gabriela Spencer-Bonilla, Laura Larrea, Katherine E. Nowakowski, Victor M. Montori, Jon Tilburt

**Affiliations:** 1 Knowledge and Evaluation Research Unit, Mayo Clinic, 200 First Street SW, Rochester, MN 55905 USA; 2 Mayo Clinic Graduate School of Biomedical Sciences, Mayo Clinic, 200 First Street SW, Rochester, MN 55905 USA; 3 Robert D. and Patricia E. Kern Center for the Science of Health Care Delivery, Mayo Clinic, 200 First Street SW, Rochester, MN 55905 USA; 4 Division of Health Care Policy and Research, Department of Health Sciences Research, Mayo Clinic, 200 First Street SW, Rochester, MN 55905 USA; 5 Division of Epidemiology, Department of Health Sciences Research, Mayo Clinic, 200 First Street SW, Rochester, MN 55905 USA; 6 Mayo Clinic School of Medicine, Mayo Clinic, 200 First Street SW, Rochester, MN 55905 USA; 7 School of Medicine, University of Puerto Rico Medical Sciences Campus, San Juan, PR 00936 USA; 8 Stritch School of Medicine, Loyola University, 2160 South First Avenue, Maywood, IL 60153 USA; 9 Division of Endocrinology, Diabetes, Metabolism, and Nutrition, Department of Internal Medicine, Mayo Clinic, Rochester, MN 55905 USA; 10 Division of General Internal Medicine, Mayo Clinic, 200 First Street SW, Rochester, MN 55905 USA; 11 Biomedical Ethics Scholars Training Program, Mayo Clinic, 200 First Street SW, Rochester, MN 55905 USA; 12 Biomedical Ethics Research Program, Mayo Clinic, 200 First Street SW, Rochester, MN 55905 USA; 13 Division of Complementary and Integrative Medicine, Mayo Clinic, 200 First Street SW, Rochester, MN 55905 USA; 14 Present address - Center for Pharmacy Innovation and Outcomes, Geisinger, 190 Welles Street, Suite 128, Forty Fort, PA USA

**Keywords:** Shared decision making, OPTION, Recording, Assessment, Communication, Cancer

## Abstract

**Background:**

How non-verbal data may influence observer-administered ratings of shared decision making is unknown. Our objective for this exploratory analysis was to determine the effect of mode of data collection (audio+video vs. audio only) on the scoring of the OPTION^5^ instrument, an observer rated measure of shared decision making.

**Methods:**

We analyzed recordings of 15 encounters between cancer patients and clinicians in which a clinical decision was made. Audio+video or audio only recordings of the encounters were randomly assigned to four trained raters, who reviewed them independently. We compared the adjusted mean scores of audio+video and audio only.

**Results:**

Forty-one unique decisions were identified within the 15 encounters. The mean OPTION^5^ score for audio+video was 17.5 (95% CI 13.5, 21.6) and for audio only was 21.8 (95% CI 17.2, 26.4) with a mean difference of 4.28 (95% CI = 0.36, 8.21; *p* = 0.032).

**Conclusion:**

A rigorous and well established measure of shared decision making performs differently when the data source is audio only. Data source may influence rating of observer administered measures of shared decision making. This potential bias needs to be confirmed as video recording to examine communication behaviors becomes more common.

**Electronic supplementary material:**

The online version of this article (10.1186/s12913-018-3329-x) contains supplementary material, which is available to authorized users.

## Background

Shared decision making (SDM) is an approach to clinical deliberation in which patients and clinicians think, talk, and feel their way through a troubling and uncertain situation. Together, they settle on a course of action that is sensitive to and respectful of both the research evidence and the patient’s values and context [1]. SDM has been called the “pinnacle of patient centered care” [2] and has begun to be incorporated into clinical practice guidelines [3, 4].

As SDM is incorporated into clinical practice guidelines the need to understand and measure whether and to what extent it is occurring becomes essential. Several different approaches to measuring SDM have been proposed [5–8]. These approaches differ based on which viewpoint is being assessed. Some measures, such as CollaboRATE [9, 10], the decisional conflict scale [11], and the 9-item SDM questionnaire (SDM-Q-9) are self-reported from the patient’s perspective [12], while others are self-reported from the clinician perspective such as the SDM-Q-Doc [13]. Another approach is to have a third party not involved in the encounter assess the occurrence of SDM. One such measure is the 5-item Observer OPTION^5^ instrument [14, 15]. The five items are designed to capture plausible mediating behaviors associated with SDM: *drawing attention to the existence of options, supporting the patient through the process of information sharing and deliberation, sharing information, eliciting preferences, and integrating preferences* [15]. As it is a third party measure, the data to which this measure is applied (i.e. the clinical encounter) is often captured either through audio or video recording [16]. Visual data may affect ratings of communication by showing non-verbal behavior that may affect observer ratings of clinician communication behavior [17]. Existing literature, however, is mixed on the effects of different modes of data collection on the subsequent analysis of data [18–23]. Understanding the effect of different modes of data collection on subsequent ratings of SDM is important because it may explain both within and across study differences in ratings of SDM. It may also aid in identifying best methodological practices for researchers.

This exploratory pilot study aimed to determine the effects of data mode, examining audio+video vs. audio only, on OPTION^5^ scores.

## Methods

### Ethical approval

This study was reviewed and approved by the University of Southern California School of Medicine and Mayo Clinic Institutional Review Boards.

### Data source

**Fig. 1:**
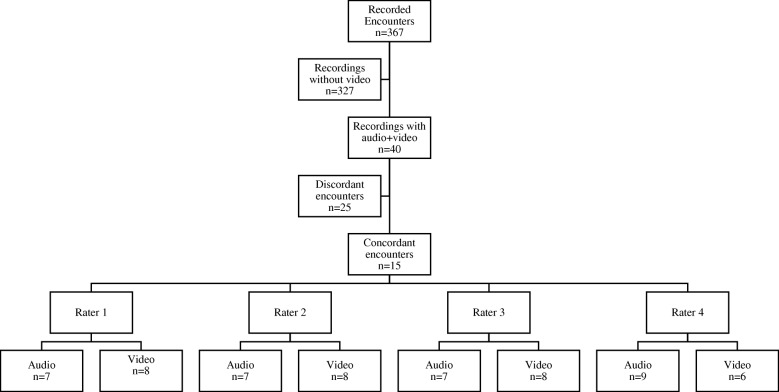
Study flow diagram

All data for this study came from an observational study on cancer communication [24]. In that study, patients were approached just prior to their regularly scheduled oncology encounter at a single tertiary cancer center in the Midwest United States and consented for participation. A study coordinator placed a small digital audio recorder in the exam room for all participants (*n* = 367), turning it on immediately before the encounter and off immediately after. For the final 40 of these encounters, the study coordinator placed a small video recorder in the room instead of a digital audio recorder. Among these 40 encounters we identified 15 in which both the patient and clinician agreed (through survey rating) that a clinical decision was made [25]. We used recordings from these 15 encounters to conduct this exploratory analysis (Fig. 1**).**

### Procedure

First, a team of two raters met to review a pilot set of encounters not included in the study sample and to develop criteria and consensus of what should be considered a “decision” for the purposes of comparative rating. To be counted as a decision, the patient-clinician discussion had to focus on issues specifically related to the potential medical management of the patient and two or more alternatives had to be presented or available. A single rater (KEN) then listened to all recorded encounters and flagged all decisions that met these criteria; multiple decisions could be identified in a single encounter.

All decisions identified by this procedure were then coded by four additional trained raters (GS-B, LL, BK, CF) with the OPTION^5^ instrument. Three raters (GS-B, LL, BK) had training in medicine and one rater had experience in study coordination (CF). Three raters also worked in a SDM research group (GS-B, LL, CF), while the fourth worked in a bioethics research group (BK). The Observer OPTION^5^, a five item measure, rates decisions on a scale from 0 to 4, with 0 representing “no effort” and 4 representing an “exemplary effort” to exhibit each of the five mediating behaviors [26]. We summed the score from each item and re-scaled to a normalized range from 0 to 100 (e.g. a score of 2 on each of the 5 items would result in a summed score of 10/20, which would be re-scaled to 50/100).

### Study design

Four raters were randomly assigned to audio only or audio+video conditions for each of the 15 encounters. When raters were assigned the audio only condition, they turned off the video input or otherwise did not view the video input. The statistician, blinded to rater identity, assigned reviewers’ files in a systematic way to ensure that all raters reviewed unique encounters from each mode (audio+video or audio only). For any given encounter recording, a rater reviewed either the audio only or the audio+video recording. Each of the 30 recordings (i.e., 15 audio only and 15 audio+video) were coded independently by two raters.

Prior to scoring any decisions or encounters, raters were trained in the use of OPTION^5^ using an online training module created by the developers of the instrument and clarified any remaining questions directly with the developers. Raters were also provided the scoring manual [26] created by the developers of OPTION^5^ as well as an investigator-developed protocol (Additional file 1) to the scoring of decisions using OPTION^5^. To minimize rater variability, the four raters calibrated their coding using a set of three encounters (audio+video) not included in the study dataset and prior to this experiment.

### Statistical analysis

To assess the impact of recording mode on OPTION^5^ scoring, we used a generalized hierarchical model in which the fixed effects were the mode (audio+video vs. audio only) and reviewer and the random effects were the encounter and clinician. This approach accounts for an encounter having multiple decisions, clinicians having multiple encounters, and assumes the error about the effect on OPTION^5^ within an encounter can vary from encounter to encounter and clinician to clinician. To estimate the average OPTION^5^ score per recording mode, the predictive margins were calculated providing an adjusted average score with 95% confidence intervals [27]. The predictive margins allowed us to isolate the impact of the mode (audio+video or audio only) by taking the adjusted model with all values and setting mode to either audio+video or audio only. This allowed us to find the average mean effect per mode to determine the amount of difference in a more quantifiable outcome. The statistical analysis was conducted using Stata version 14.0 (College Station, TX).

## Results

The mean patient age was 64, a majority of patients had at least some college, and 53% of patients were female (Table 1). Of the 8 clinicians who participated in the 15 encounters, the median number of encounters per clinician was 2 with a range of 1 to 3.

**Table 1:** Encounter Characteristics

	*N* = 15
Patient Female: *N* (%)	8 (53.3%)
Patient Age: Mean (SD), Range	63.7 (16.9), (28, 90)
Patient Race – White: *N* (%)^a^	14 (93.3%)
Patient Education^a^: High school or less	6 (40%)
Some College or Vocational	4 (26.7%)
4 Year College Degree	3 (20.0%)
Post Graduate Degree	1 (6.7%)
Annual Household Income(US Dollars)^b^: <$35,000	3 (20%)
$35 to < $50,000	2 (13.3%)
$50 to < $75,000	4 (26.7%)
$75,000+	3 (20%)
Clinician Count: *N*, Median Number of Encounters (Range)	8, 2 (1, 3)

^a^Missing response for 1 patient, included in percentages

^b^Missing responses for 3 patients, included in percentages

Forty-one unique decisions were identified within the 15 encounters. Ten of the encounters included more than one decision with a maximum of six decisions occurring in two encounters (Additional file 2: Table S1). These ranged from specific decisions such as choosing between radiation or chemotherapy, to less specific discussions such as “what to do next” ((Additional file 3: Table S2).

**Fig. 2:**
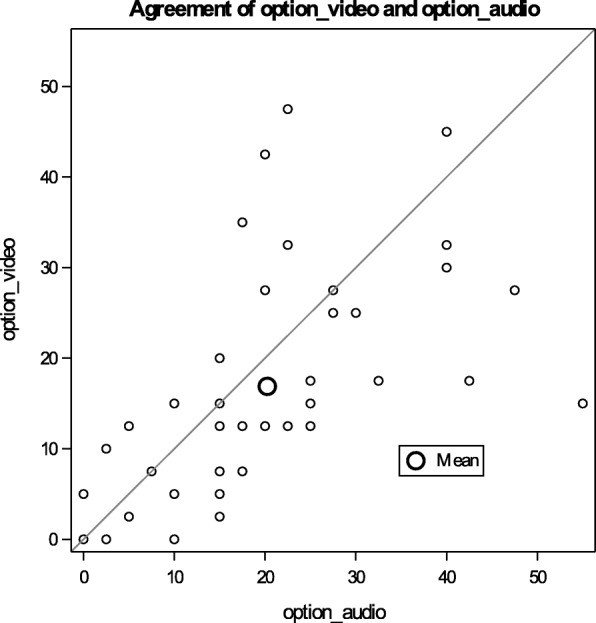
OPTION^5^ score agreement between audio+video and audio only

The overall mean OPTION^5^ score for the audio only recordings was 21.8 (95% CI 17.2, 26.4) out of 100. For the audio+video recordings, the mean score was lower (17.5, 95% CI 13.5, 21.6). Thus, the average adjusted OPTION^5^ score for audio only recordings was 4.3 points higher than audio+video with a 95% CI of 0.36, 8.21. This overall difference in mean OPTION^5^ score was reflected in the mean scores for four of the five constituent items of the OPTION^5^ instrument (Table 2). For all items, except for item 2 (i.e. clinician supports the patient through the decision making process), audio only was associated with higher scores to varying degrees (i.e. on average, audio is 0.04 points higher for item 4 – eliciting preferences, to 0.31 points higher for item 3 – sharing information). Visual inspection of a plot of total OPTION^5^ scores for the audio+video mode (y-axis) versus scores for the audio only mode (x-axis) (Fig. 2) suggests that at the higher ranges of OPTION^5^ scoring, there appears to be greater heterogeneity between audio+video and audio only scores, than on the lower end of OPTION^5^ scoring. The concordance between reviewers within mode (i.e. audio+video or audio) was approximately 60% (audio: 58.3 95% CI (37.7, 78.9); audio+video: 60 95% CI (40.4, 79.6)).

**Table 2:** OPTION^5^ scores – audio+video vs. audio only

	Mean (95% CI)	Audio+Video: Mean (95% CI)	Mean Difference (95% CI)	*P*-Value
OPTION^5^ Total: (0–100 scale)	21.8 (17.2, 26.4)	17.5 (13.5, 21.6)	4.28 (0.36, 8.21)	0.032
OPTION^5^ Question 1: (0–4 scale) Draw attention to options	1.22 (0.96, 1.49)	0.95 (0.68, 1.22)	0.27 (0.03, 0.52)	0.028
OPTION^5^ Question 2: Support the patient	0.35 (0.16, 0.55)	0.38 (0.19, 0.57)	−0.03 (− 0.17, 0.12)	0.74
OPTION^5^ Question 3: Share information	1.22 (0.94, 1.5)	0.91 (0.63, 1.18)	0.31 (0.08, 0.55)	0.009
OPTION^5^ Question 4: Elicit preferences	0.70 (0.46, 0.95)	0.66 (0.42, 0.91)	0.04 (−0.22, 0.30)	0.76
OPTION^5^ Question 5: Integrate preferences	0.83 (0.61, 1.06)	0.66 (0.43, 0.89)	0.17 (−0.09, 0.44)	0.20

Predictive margins calculated and adjusted by the fixed effect of reviewer and the random effect of clinician and encounter

## Discussion

This small methodological experimental sub-study embedded in a larger observational study demonstrates that mode of recorded data, audio+video vs. audio only, influences ratings of a prominent and commonly used third-party measure of SDM, the OPTION^5^ instrument.

This finding is similar to those reported for other measures of communication in healthcare [18–22]. Riddle et al. [22] found that when clinical encounters were coded using the Moffitt Accrual Analysis System (MAAS) and the Roter Interaction Analysis System (RIAS) there were significant differences (*p* < 0.028 for MAAS and *p* < 0.01 for RIAS), between audio and video coding. In contrast to this study however, other studies have failed to show a difference between audio only and audio+video modes when rating aspects of health communication. Nicolai et al. [19] measured empathic communication during clinical encounters using the Rating Scales for the Assessment of Empathic Communication in Medical Interviews; they found no difference between audio and video formats for rating empathy but both modes resulted in higher empathy scores over transcripts. Williams et al. examined video vs. audio only clips of nursing care and rated them using the Emotional Tone Rating Scale and found that the modes were highly correlated and only one item (patronizing) after adjustment for multiple comparisons, was significantly different between modes (audio scored higher). Weingarten et al. [20]. assessed clinical encounters for patient-centeredness using the Henbest and Stewart scale of patient-centeredness and found no difference (both mean scores 1.94 out of 3) between audio and video. Dent et al. [18] assessed communication in simulated cancer consultations using the Cancode interaction analysis system and found substantial agreement [28] between audio and video as indicated by kappas of 0.72 and 0.77 for the function and content dimensions of Cancode, respectively [18]. The discrepancies in the literature on the effect of mode could reflect the different constructs being measured, with some more affected by the additional information contained in audio+video (e.g. non-verbal behaviors). In a review of the impact of clinicians’ personality and interpersonal behaviors on the quality of care, Boerebach et al. found mixed results for the impact of non-verbal behaviors on patient reported ratings of quality of care [17]. It is therefore plausible that clinicians’ non-verbal behavior affected third party’s ratings and for example, may explain the non-statistically significantly higher score for item 2. However, the difference we found was driven by items which coded for sharing information and drawing attention to the existence of options; items which arguably are more objective as compared to supporting the patient through the process of information sharing and deliberation. These findings, if confirmed in larger samples, could more definitively establish the relationship between data collection mode and OPTION^5^ scoring. Ideally, confirmatory studies will be conducted using mixed methods including interviews with raters so that differences could be further explored and explained.

This study has several limitations. Our sample was drawn from cancer consultations with no uniform focus of the discussion. This is in contrast to the typical use of the OPTION^5^ instrument, which is usually used to score a specific, focused decision (e.g. decisions about medication treatments for diabetes or management strategies for heavy menstrual bleeding). As a result, many discussions with low scores could have created an artificial floor effect, thus limiting our ability to detect a difference. We detected a difference and therefore, the effect of an artificial floor was limited. Our analytic choice to pool all decisions may have differentially affected our mode comparison (i.e. audio+video vs. audio only), thus potentially creating a difference between modes which may be instead due to aspects of the decisions or discussions.

Whether the effects observed in our exploratory study constitute a bias is an important question. Arguably, what matters is how the actors in the encounter experienced it. Third-party measures seek to ascertain second-hand how that process went. It offers an important degree of detached objectivity helpful for documenting verifiable behaviors that may not be easily recalled by participants. Video offers the advantage of non-verbal inputs, yet the meaning of those inputs, largely constituted by gesture, posture, and eye contact and other non-verbal behaviors are as influenced by experience and culture as much or more as the spoken word. Thus, we cannot say whether these differences are good or bad. However, at a minimum, they clearly have implications for aggregating outcome data across trials where a variety of SDM measures and observer-administered ratings are used. They also may influence effect sizes estimation for design of future trials.

While our results merit further investigation on methodologic grounds, investigators studying SDM and patient-clinician communication need to consider the pragmatic implications of choosing audio or audio+video to record encounters. For example, some patients may be more comfortable with audio only recording as it is less intrusive and offers a greater degree of privacy. Yet, this choice limits the richness of the available data for the investigator as audio+video provides additional data such as non-verbal communication which may provide important contextual and non-verbal relational information not available with audio only recordings. Investigators planning to record encounters need to consider these trade-offs and the extent they matter for a given set of study objectives.

## Conclusion

Shared decision making is an important component of patient centered care [2, 29]. Research, quality assurance, and quality improvement should be supported by accurate and reliable measures. Different ways of measuring SDM are available including third party measures such as the OPTION^5^ instrument. Due to patient preference, available resources, or clinician comfort level discussions may be recorded as video or audio only. This study suggests that, for the OPTION^5^ instrument, mode of recording affects the rating of SDM in the clinical encounter. Additional research is needed to confirm and further understand the effects of mode of recording on ratings of SDM across of variety of conditions and decisions.

## Additional files

Additional file 1: Preparing to Score OPTION^5^. This is the investigator developed protocol for scoring OPTION5 (DOCX 14 kb)

Additional file 2: **Table S1.** Number of discussions within encounters. This Table lists and enumerates the number of discussions that occurred within encounters in our dataset. (DOCX 13 kb)

Additional file 3: **Table S2.** Discussion Topics. This Table lists and enumerates the topics of discussions that were present in the encounters within our dataset (DOCX 14 kb)

## Abbreviations

IRB: Institutional review board
MAAS: Moffitt accrual analysis system
RIAS: Roter interaction analysis system
SDM: Shared decision making
