# Supplementary material for: Comparison of audio vs. audio + video for the rating of shared decision making in oncology using the observer OPTION5 instrument: an exploratory analysis
Source: BMC Health Serv Res. 2018 Jul 4;18:522. doi: 10.1186/s12913-018-3329-x (PMC6033223; doi:10.1186/s12913-018-3329-x)
Supplement: Supplementary file 1 — Preparing to Score OPTION5. This is the investigator developed protocol for scoring OPTION5 (DOCX 14 kb) [file 12913_2018_3329_MOESM1_ESM.docx]

**Preparing to score OPTION^5^**

1. Before scoring OPTION^5^ be sure to have the scoring manual at hand to help guide your scoring and remind you of the wording of the 5 items that comprise OPTION^5^. If you do not have a copy of the manual, you can find a copy on the website where you trained to use OPTION5. Alternatively, you can e-mail Michael Gionfriddo ([Gionfriddo.michael@mayo.edu](mailto:Gionfriddo.michael@mayo.edu)) for a copy.
2. For this study you will be scoring videos. Some you will score while watching the visual component and listening to the audio component, others you will score only listening to the audio component. In the audio/videos you are assigned you will score each decision being made in that consultation, noting that there will be multiple decisions per consultation. This means that for some decisions a clinician may do a good job supporting the patient in the decision making process and deliberating about options, but for others they may not do such a good job. This is OK and to be expected.
3. While the data may be collected in an electronic format (e.g. REDcap), it is helpful to have a hardcopy of the scale to score on. This helps in a few ways: 1) it provides a back-up if there is an error in electronic data entry or if the electronic database crashes 2) should conflicts arise, I have found that having hard copy versions facilitates discussion better than electronic data collection forms 3) Hard copies facilitate scoring, especially since you will be watching/listening on a computer and jumping between windows may not be efficient. Copies of the OPTION5 scoring sheet can be found on the website where you trained to use OPTION5. Alternatively, you can e-mail Michael Gionfriddo ([Gionfriddo.michael@mayo.edu](mailto:Gionfriddo.michael@mayo.edu)) for a copy.
4. An important component of this study is examining whether or not encounters are scored differently based on whether either audio or video is available. Therefore, if you are assigned a video be sure to actually WATCH the video and NOT just LISTEN to it.
5. Therefore, regardless of whether you are scoring a video or audio only file, it is advisable that you score as you go along. One way to think about this is that at the beginning all items are rated 0 and as the clinician or patient performs a behavior consistent with the item being scored, the score increases.
6. You are scoring decisions, there may be many decisions per encounter, be sure to have separate score sheets for each decision.
7. When you do hear a behavior that you believe should be scored positively it is advisable to pause the video/or audio so that you can: 1) mark down the time you heard the behavior for auditing/calibration purposes 2) focus on scoring the item and not miss additional behaviors while scoring the item 3) Have the opportunity to write down your thoughts about why you scored an item a certain way 4) gives you time to switch between decision score sheets as decision making may not occur linearly (i.e. decision 1 🡪 decision 2 🡪 decision 3; rather more like decision 1 🡪 decision 2🡪 decision 1🡪 decision 3 🡪 decision 1 🡪 decision 3 🡪 decision 2, etc.)
8. When you reach the end of a video review your score sheets for each video to: 1) ensure that all items received a score (given the strategy above it may be advisable to investigate/think about any scores of 0 to make sure they weren’t present) 2) Make sure you feel confident about your scores given the comments you have made along the way (if you do not feel confident or wish to check again this is where time-stamping behaviors becomes helpful).
9. If you have any questions about OPTION5, please contact Michael Gionfriddo ([Gionfriddo.michael@mayo.edu](mailto:Gionfriddo.michael@mayo.edu))
