# Supplementary material for: Comparison of audio vs. audio + video for the rating of shared decision making in oncology using the observer OPTION5 instrument: an exploratory analysis
Source: BMC Health Serv Res. 2018 Jul 4;18:522. doi: 10.1186/s12913-018-3329-x (PMC6033223; doi:10.1186/s12913-018-3329-x)
Supplement: Supplementary file 2 — Table S1. Number of discussions within encounters. This Table lists and enumerates the number of discussions that occurred within encounters in our dataset. (DOCX 13 kb) [file 12913_2018_3329_MOESM2_ESM.docx]

Supplemental Table 1: Number of discussions within encounters

| Number of Discussions | N |
| --- | --- |
| 1 | 15 |
| 2 | 10 |
| 3 | 7 |
| 4 | 4 |
| 5 | 3 |
| 6 | 2 |
