# Supplementary material for: Comparison of audio vs. audio + video for the rating of shared decision making in oncology using the observer OPTION5 instrument: an exploratory analysis
Source: BMC Health Serv Res. 2018 Jul 4;18:522. doi: 10.1186/s12913-018-3329-x (PMC6033223; doi:10.1186/s12913-018-3329-x)
Supplement: Supplementary file 3 — Table S2. Discussion Topics. This Table lists and enumerates the topics of discussions that were present in the encounters within our dataset (DOCX 14 kb) [file 12913_2018_3329_MOESM3_ESM.docx]

Supplemental Table 2: Discussion topics

| **Discussion Topic** | **N (%)** |
| --- | --- |
| What should be done? | 4 (9.8%) |
| When to do follow up? | 1 (2.4%) |
| Where to do follow up? | 2 (4.9%) |
| Radiation vs. Chemo | 3 (7.3%) |
| Modify Chemo (reduce?) | 1 (2.4%) |
| Treating Symptoms | 9 (22.0%) |
| What to do about worsening symptoms? | 5 (12.2%) |
| Dealing with cancer stress | 1 (2.4%) |
| Chemo | 1 (2.4%) |
| Biopsy | 1 (2.4%) |
| Putting in a port | 1 (2.4%) |
| Cancer Treatment | 2 (4.9%) |
| Body scans | 1 (2.4%) |
| Draining the heart | 1 (2.4%) |
| Continuing Chemo | 2 (4.9%) |
| What are the next steps? | 4 (9.8%) |
| What if new cancer appears? | 2 (4.9%) |
